# Preparation and Characterization of Fish Skin Collagen Material Modified with β-Glucan as Potential Wound Dressing

**DOI:** 10.3390/ma14061322

**Published:** 2021-03-10

**Authors:** Marta Michalska-Sionkowska, Oliwia Warżyńska, Beata Kaczmarek-Szczepańska, Krzysztof Łukowicz, Anna Maria Osyczka, Maciej Walczak

**Affiliations:** 1 Department of Environmental Microbiology and Biotechnology, Faculty of Biological and Veterinary Sciences, Nicolaus Copernicus University in Toruń, 87-100 Toruń, Poland; oliwia.warzynska@vp.pl (O.W.); walczak@umk.pl (M.W.); 2 Department of Biomaterials and Cosmetics Chemistry, Faculty of Chemistry, Nicolaus Copernicus University in Toruń, 87-100 Toruń, Poland; beata.kaczmarek@umk.pl; 3 Department of Biology and Cell Imaging, Faculty of Biology, Institute of Zoology and Biomedical Research, Jagiellonian University, 30-387 Kraków, Poland; krzysztof.lukowicz@uj.edu.pl (K.Ł.); anna.osyczka@uj.edu.pl (A.M.O.)

**Keywords:** collagen, β-glucan, HaCaT cells, wound dressing, natural polymers

## Abstract

Collagen possesses unique properties, e.g., biocompatibility, biodegradability, and non-toxicity. However, collagen material degrades too quickly and has low mechanical properties. One of the methods of polymers’ modification is mixing them to obtain blends. In this study, the influence of β-glucan for collagen material was analyzed. The interaction between the functional groups of the polymer was analyzed by ATR-FTIR (attenuated total reflection-fourier transform infrared) spectroscopy. The influence of β-glucan on mechanical properties was evaluated. The surface properties of materials were assessed using contact angle measurements and the topography of materials was evaluated by AFM (atomic force microscope). The structure of materials was analyzed according to SEM (scanning electron microscopy) pictures. Moreover, the DPPH-free radicals’ scavenging ability and biocompatibility against erythrocytes and HaCaT cells were evaluated. Collagen and β-glucan were bound together by a hydrogen bond. β-glucan addition increased the roughness of the surface of the film and resulted in a more rigid character of the materials. A small addition of β-glucan to collagen provided a more hydrophilic character. All the materials could swell in in vitro conditions and showed antioxidant activity. Materials do not cause erythrocyte hemolysis. Finely, our cytotoxicity studies indicated that β-glucan can be safely added at small (10% or less) quantity to collagen matrix, they sufficiently support cell growth, and the degradation products of such matrices may actually provide some beneficial effects to the surrounding cells/tissues.

## 1. Introduction

Collagens are a group of major structural proteins. Currently, 28 types of collagen are known. The type of collagen depends on its structure, function, and tissue distribution [1]. Collagen (Coll) type I is the most abundant collagen in the mammalian body and constitutes the main component of the connective tissue, bone, skin, and tendons [2]. So far, the main sources of collagen for industrial applications are bovine and pig’s skins and bones [3]. However, their application has been restricted due to the outbreak of some diseases, such as foot and mouth disease (FMD) and bovine spongiform encephalopathy (BSE), which may be transferred to humans [4]. Moreover, those sources of collagen are not acceptable in research by some cultures [5]. By-products and leftovers generated by the seafood processing may be valuable sources of macromolecules, e.g., proteins, lipids, and polysaccharides. Therefore, fish collagen is of great interest to the cosmetic, pharmaceutical, and medical industries. Fish skin may be a promising source of collagen. The use of by-products from a local fish industry may decrease collagen cost and it allows to mitigate environmental problems [6]. On average, solid waste from fish processing comprises 50% of raw original material (depending on the level of processing and type of fish) [7]. The skin and bones comprise 30% of these wastes [8]. Fish skins and bones may be successfully used to isolate collagen with a 36–54% recovery rate [9]. Moreover, collagen isolated from fish waste fulfils religious requests like halal and kosher [10]. Collagen, due to its characteristics, is applied as a biomaterial for tissue engineering and wound dressings [11]. It was found that fish collagen induced keratinocyte differentiation, which is required for the formation of integrated epidermis, probably by the synergetic effects of the amino acids on stimulating the HaCaT cells’ differentiation [12]. To improve the properties of collagen-based materials, it can be mixed with other polymers [13]. 

Glucans are polysaccharides built of glucose units linked by α- (alpha) and β (beta)-type glycosidic bonds [14]. Their biological activity depends on the primary structure, molecular weight, source, and extraction method [15]. β-glucan may be isolated from different sources, like yeast, fungi, algae, edible mushrooms, and cereal grains such as oats, barley, wheat, and rye. For extraction of β-glucan from natural sources, water extraction, alkaline extraction, acid extraction, and enzymatic extraction may be used. The yield and purity of β-glucan depend on the methods employed for the extraction [16]. It was shown that β-glucan enhances wound healing by stimulating tissue granulation, human dermal fibroblast collagen biosynthesis and deposition, and, finally, re-epithelialization [17]. Moreover, β-glucan (BG) is known to stimulate macrophages in the inflammation phase of healing, enhancing their phagocytosis and secretion of chemokines and promoting the formation of new tissue [18]. β-glucan can form a crosslinking network by hydrogen bonds, increasing rigidity or gelling properties [19].

Wound dressing materials (Brennan Medical, Inc, St. Paul, MN, USA) with collagen and β-glucan were already studied by Delatte et al. in 2001 [18]. However, the authors studied commercially available wound dressing BGC MATRIX® and its effect on patients’ health. They showed that this commercial wound dressing simplifies wound care and significantly decreases post-injury pain. In our research, we prepared polymeric sheets based on collagen obtained from fish skin and modified by β-glucan and characterized their physicochemical and biological properties for potential wound dressing. 

## 2. Materials and Methods

### 2.1. Collagen Isolation 

Collagen was obtained in our laboratory from *Hypophthalmichthys nobilis* skin. The skins were sourced as by-products from the local fish industry—they are waste. Collagen was obtained using the method described in our previous publication [20]. First, soft tissue was removed from the skin. The skins were cut into 2 cm × 2 cm squares and cleaned with distilled water. The skin was extracted with 0.1 M NaOH for 4 days to remove non-collagenous proteins. Then, fat was extracted with 10% butyl alcohol for 1 day and washed with distilled water. The insoluble matter was extracted with 0.5 M acetic acid for 2 days and protein was salted out by adding NaCl (to a final concentration of 2 M). Precipitated collagen was centrifuged (Rotina 38R, Hettich, Tuttlingen, Germany) at 10,000 rpm for 30 min. The resulting precipitate was dissolved in 0.5 M acetic acid, dialyzed (MWCO = 12–14 kDa, Serva, Heidelberg, Germany) against 0.1 M acetic acid for 5 days, changing the solution every 24 h. Then, the solution was frozen (−18 °C) and lyophilized (ALPHA 1–2 LD plus, CHRIST, Osterode am Harz, Germany). Collagen was stored at −18 °C.

### 2.2. Materials Preparation

One percent (1%) (*w*/*v*) collagen solution was prepared with 0.1 M acetic acid as a solvent, and 5% β-glucan (oat 1–3 β-glucan, cosmetics ingredient) solution was prepared in deionized water and heat in a water bath without boiling. The solutions were mixed together in collagen/β-glucan ratios of: 100/0, 90/10, 70/30, and 50/50 (*w*/*w*). The materials were obtained by the solvent casting method. 

### 2.3. ATR-FTIR (Attenuated Total Reflection-Fourier Transform Infrared) Spectroscopy

The structure of collagen and β-glucan as well as the interaction between them were confirmed by attenuated total reflection infrared spectroscopy using Nicolet iS10 equipment (Shimadzu, Kyoto, Japan). All the spectra were recorded by absorption mode at 4 cm^−1^ intervals and 64-times scanning [21].

### 2.4. Mechanical Properties

Mechanical properties of films were tested by using the Zwick and Roell testing machine (Ulm, Germany). Materials were cut into bog-bone shapes of initial dimensions of 50 mm in length and 4 mm in width. The thickness of the samples was determined using an ultra-meter type A-91 (producer: Manufacture of Electronic Devices, Warsaw, Poland). Parameters of the testing program: 200 mm/min speed starting position, 0.1 MPa initial force, and 5 mm/min the speed of the initial force. For each kind of film, at least five samples were tested. Results were prepared as average values with standard deviation (SD). 

### 2.5. Surface Properties

The topographic structure of the polymeric films was observed using an atomic force microscope (AFM, Veeco Metrology, Inc., Santa Barbara, CA, USA). Images were obtained using a multimode scanning probe microscope with a Nanoscope IIIa controller (Digital Instruments, Santa Barbara, CA, USA) operating in the tapping mode, in air, at room temperature. Surface images were acquired at fixed resolution (512 × 512 data points) with a scan rate of 1.97 Hz. Silicon tips with spring constant of 2–10 N/m were used. Roughness parameters were calculated from a 10 µm × 10 µm scanned area using Nanoscope software (v6.11, Bruker Optoc GmbH, Ettlingen, Germany) [22].

### 2.6. Scanning Electron Microscopy (SEM)

The cross-section of the materials was imaged using a Scanning Electron Microscope (LEO Electron Microscopy Ltd., Cambridge, UK), and the samples were covered by gold particles. Images were recorded at 2500 times of magnification and the voltage used for the acquisition was 300 kV. 

### 2.7. Contact Angle Measurements

For this analysis, the materials were prepared on microscopic slides. Contact angle measurements were performed using a goniometer equipped with a system of drop shape analysis (DSA 10 Control Unit, Krüss, Hamburg, Germany) for two measurement liquids. Because the prepared materials swell in water, glycerol was used as a hydrophilic liquid. Diiodomethane was chosen as a hydrophobic liquid. Surface free energy and its polar and dispersive components were calculated using the Owens-Wendt method [22].

### 2.8. Antioxidant Properties

To assess the antioxidant properties of the prepared materials, DPPH reagent (2,2-Diphenyl-1-picrylhydrazyl, free radical, 95%) (Alfa Aesar, Kandel, Germany) was used. For the reaction, 250 µM of DPPH in methanol was prepared. The materials were cut into 1 cm^2^ pieces and placed in a 24-well plate filled with 1500 µL of DPPH. The samples were incubated at room temperature without access to light for 1 h. The absorbance was measured using a spectrophotometer (Hitachi U-1900, Tokyo, Japan) at a wavelength of 517 nm. The antioxidant ratio was calculated using the equation:

$$Antioxidant\;ratio\;\left[\%\right]=\frac{Abs_{DPPH}-Abs_{sample}}{Abs_{DPPH}}*100$$

where *Abs_DPPH_* is an absorbance of DPPH solution, and *Abs_sample_* is an absorbance of DPPH solution after incubation with the materials.

The analysis was performed in triplicate. The results were presented as mean values with standard deviation. 

### 2.9. Swelling Properties 

Swelling properties were evaluated according to the method described by Zare-Gachi et al. with small modifications [23]. Dried and weighed films were soaked in PBS (phosphate buffered saline) buffer (pH = 7.4) for 1 h at 37 °C. After incubation, the films were carefully taken out and excess of fluids was removed on the filter paper, and then weighed until constant weight. The swelling ratio (*Sw*) was calculated by using the equation:

$$Sw=\frac{W_{S}-W_{\;D}}{W_{D}}\times 100\%$$

where *W_S_* is the weight of the swollen film and *W_D_* is the weight of the dried film.

The films were analyzed in four repetitions and the results are presented as mean values with standard deviation. 

### 2.10. Color Change 

Each sample was cut into 2 cm × 2 cm squares and placed in a 12-well plastic holder. The droplets of three liquids were applied on their surface: 2 mol/L HCl, 2 mol/L NaOH, and deionized water. Variations of color were measured after 1 h using a colorimeter (Corneometer CL 400, Courage, Khazaka, Köln, Germany). The color parameter L (which describes the sample lightness), a (along the X axis red (+) to green (−)), b (along the Y axis yellow (+) to blue (−)) were evaluated [24]. Each sample was analyzed in triplicate and mean values were used for calculation of the total color difference (Δ*E*). The total color difference (Δ*E*) was then calculated using the equation:

$$\Delta E={\left(\Delta L^{2}+\Delta a^{2}+\Delta b^{2}\right)}^{0.5}$$

where Δ*L* = L − L_0_, Δ*a* = a − a_0_, Δ*b* = b − b_0_, and L_o_, a_o_, b_o_ are color values of the control.

### 2.11. In Vitro Blood Biocompatibility

The direct contact method was used to evaluate blood biocompatibility of the prepared materials using the methodology described by Zhou et al., with a small modification [25]. Anticoagulated whole sheep blood was used to determine the effect of materials on red blood cell hemolysis. Sheep blood (0.2 mL) was added to the physiological saline solution (10 mL) containing different specimens (1 cm^2^ area). The tubes were incubated at 37 ° C for 60 min. Then, the suspension was centrifuged at 10,000 rpm for 10 min and the absorbance of the supernatants was measured by microplate reader Multiscan FC (Thermo Fisher Scientific, Waltham, MA, USA) at 540 nm. Positive and negative samples were prepared by adding 0.2 mL of blood to distilled water and physiological saline, respectively. Samples were prepared in triplicates. Hemolysis rate was calculated using the following equation:

$$Rate\;of\;hemolysis\;\left[\%\right]=\;\frac{OD_{specimen}-OD_{negative}}{OD_{positive}-OD_{negative}}$$

### 2.12. Cytocompatibility Studies

Unless stated otherwise, human keratynocytes cell line HaCaT [26] and cell culture reagents were purchased from Thermo Fisher Scientific (Waltham, Massachusetts, USA). For cytocompatibility studies, the material sheets were cut into round disks fitting the bottom of individual wells of 24-well tissue culture plates. The disks were sterilized by soaking in 70% ethanol (water solution), then they were washed briefly with phosphate-buffered saline (PBS) to remove ethanol traces, exposed to UV (ultraviolet) light (10 min each side), and left overnight under a laminar flow to dry. A separate set of sterilized materials was used for direct cell culture and another set for the preparation of material extracts. The latter were prepared by 24 h incubation of materials in a growth medium (1 mL/well) composed of alpha-minimum essential medium (αMEM) supplemented with 10% fetal bovine serum (FBS) and antibiotics (penicillin/streptomycin 1% solution). Cells were either seeded directly on the material surface or on tissue culture plastic (TCP) at the density of 2 × 10^4^ cells/cm^2^. The cells seeded directly on the materials were assessed for the viability after 24 h culture. Cells seeded on TCP were cultured for 24 h, then exposed to material extracts for an additional 24 h and assessed for viability. The material set that was used to prepare material extracts was used to culture cells for 72 h. To assess cell viability, cultures were washed with PBS, and 0.2 mL solution of 10% MTS (3-(4,5-dimethylthiazol-2-yl)-5-(3-carboxymethoxyphenyl)-2-(4-sulfophenyl)-2H-tetrazolium, inner salt) reagent (CellTiter96Aqueous One Solution Cell Proliferation Assay; Promega) in phenol-free alpha-MEM was added to individual wells. Cells cultured on TCP without any stimulation were considered as a general reference material. The plates were incubated at 37 °C until apparent change of color from yellow to brownish. Then, the media were transferred to individual wells in 96-well plates and the absorbance was recorded at 492 nm using a plate reader. Data were analyzed by one-way analysis of variance (ANOVA) using Tukey’s post hoc tests (*p* ≤  0.05).

## 3. Results

### 3.1. ATR-FTIR Spectroscopy 

**Figure 1:**
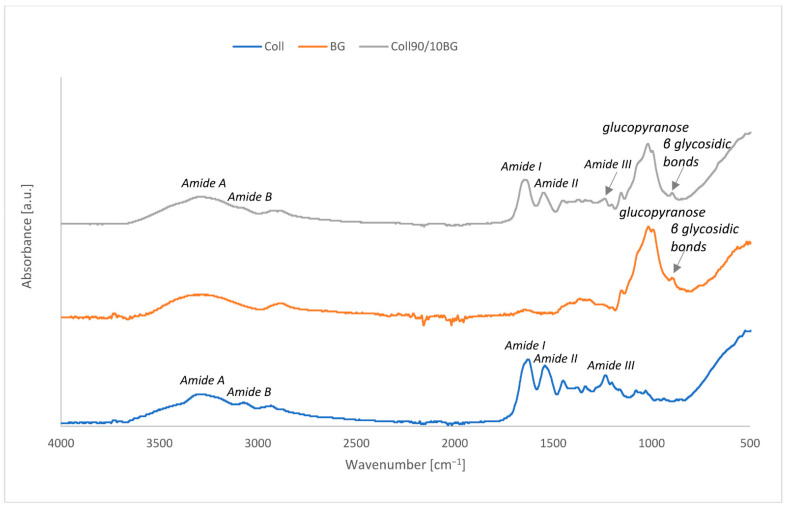
ATR-FTIR spectra collected for collagen, β-glucan, and collagen/β-glucan material.

ATR-FTIR spectroscopy was performed to confirm β-glucan present in collagen film as well as to assess the interaction between those polymers. The results obtained for collagen, β-glucan powder, and Coll90/10BG are presented in Figure 1 and assignments of the major frequencies are shown in Table 1. 

The main bonds recorded for collagen correspond to wavenumbers: 3315, 3049, 1618, 1527, and 1225 cm^−1^ respectively, and they are characteristic for collagenous samples. The absorption characteristics of Amide A, commonly associated with N–H stretching vibration, occurs in the wavenumber range of 3300–3440 cm^−1^ [13]. The maximum of the absorption peak of our collagen was found at 3315 cm^−1^. Amide B peak was found at 3049 cm^−1^. The maximum peaks found at 1618 and 1527 cm^−1^ were assigned to Amide I and Amide II, respectively. The peak at 1225 cm^−1^ corresponds to Amide III. For the analyzed β-glucan spectrum, the characteristic peak was observed at 1015 cm^−1^. The FTIR peak in the region of 928–1200 cm^−1^ (mainly due to C-C and C=O stretching vibrations) indicates the presence of polysaccharides [27]. The adsorption at 1014 cm^−1^ indicates the presence of glucopyranose moiety in oat β-glucan. The adsorption peak at 896 cm^−1^ is characteristic of β-linked glycosidic bonds [28]. Moreover, no adsorption near 840 cm^−1^ showed the absence of α-linked glycosidic bonds [29]. On Coll90/10BG spectra, all the described peaks are visible, both for collagen and β-glucan. It confirms that both polymers are present in the material sheet. Hydrogen bonds between collagen and β-glucan are formed by interaction with the hydroxyl group from the collagen chain. No additional peaks were observed, which allows to assume that covalent bonds between collagen and β-glucan were not formed. The shift of Amide A (N-H), Amide B (O-H), and Amide I (C=O) is noticed when compared to Coll with and without BG. It suggests that those groups are included in the hydrogen bond formation.

### 3.2. Mechanical Properties 

**Figure 2:**
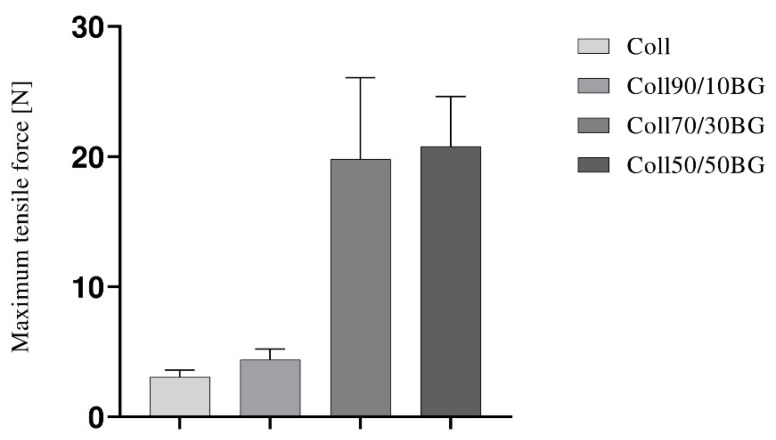
Maximum tensile force at break for the films based on collagen and β-glucan in the weight ratios: 100/00, 90/10, 70/30, and 50/50.

During mechanical testing of the samples, data on maximum tensile force at break were collected. The obtained results are presented in Figure 2.

The maximum tensile force needed to break the collagen film was 3.1 N. β-glucan addition into collagen films increases the tensile force: 10% of β-glucan into collagen materials increases maximum tensile force up to 4.4 N. Higher addition of β-glucan enhances tear-resistance and causes an increase of tensile force approximately 6.5 times. The difference between the results obtained for Coll70/30BG and Coll50/50BG was only 1 N. BG addition caused increase of the strength and reduction of the flexibility of the materials.

### 3.3. Roughness Parameters

**Figure 3:**
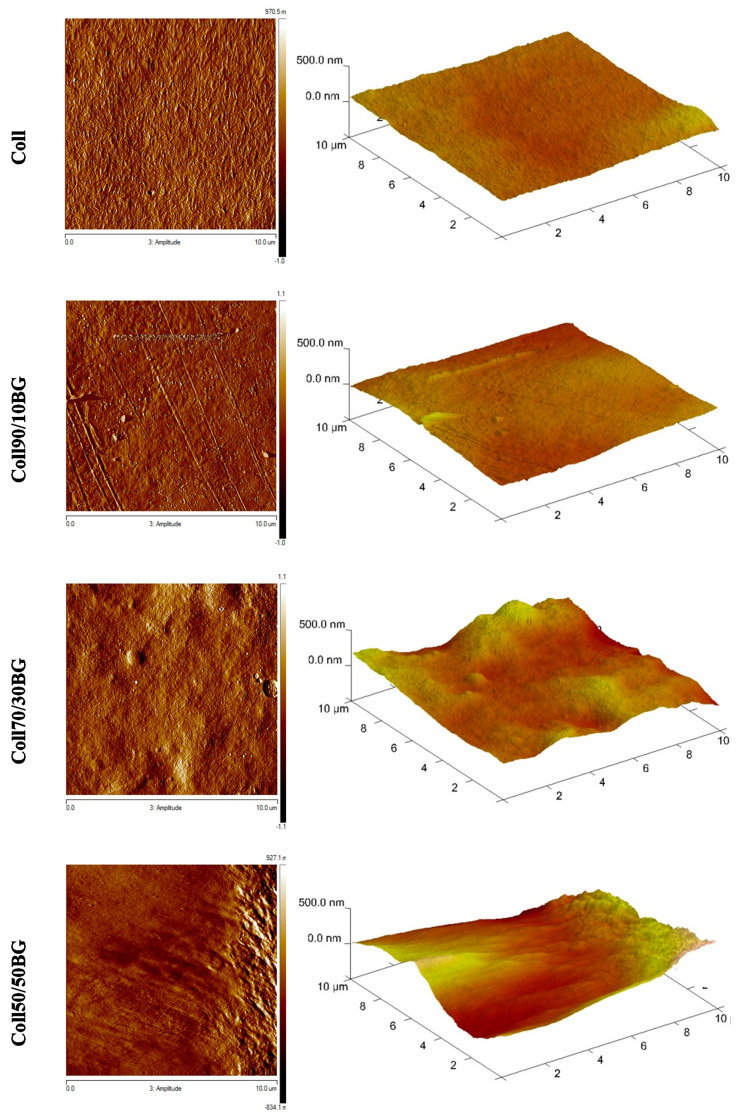
Pictures of structure morphology of collagen and collagen/β-glucan films.

The morphology of Coll/BG materials was visualized using AFM analysis. Pictures in two- and three-dimensional (2D and 3D) form are presented in Figure 3. Additional information about the roughness of the prepared materials can be found in Table 2, where the Rq and Ra values are presented. Rq is a root mean square roughness and represents the standard deviation of the distribution of surface heights. Ra is an arithmetic average height parameter defined as the average absolute deviation of the roughness irregularities from the mean line over one sampling length [22]. 

To determine the β-glucan impact on collagen material, a collagen film was chosen as a control sample. The topography of the prepared materials is homogenous. Collagen triple helical structures are not easy to detect, due to their small size compared to the high roughness of collagen [30]. It can be seen that all kinds of films are compact and non-porous. Films obtained from collagen and collagen with 10% BG addition have smoother surface compared to materials with 30% and 50% BG addition. On the surface of Coll70/30BG and Coll50/50BG, one can see micro-folds’ formation. Rq and Ra parameters increase with the increasing amount of β-glucan in the materials. Both parameters increase almost 4 times for Coll50/50BG compared to pure collagen. In Figure 3, especially for Coll90/10BG material, the random distribution of fiber-like structure was observed.

### 3.4. Scanning Electron Microscopy

**Figure 4:**
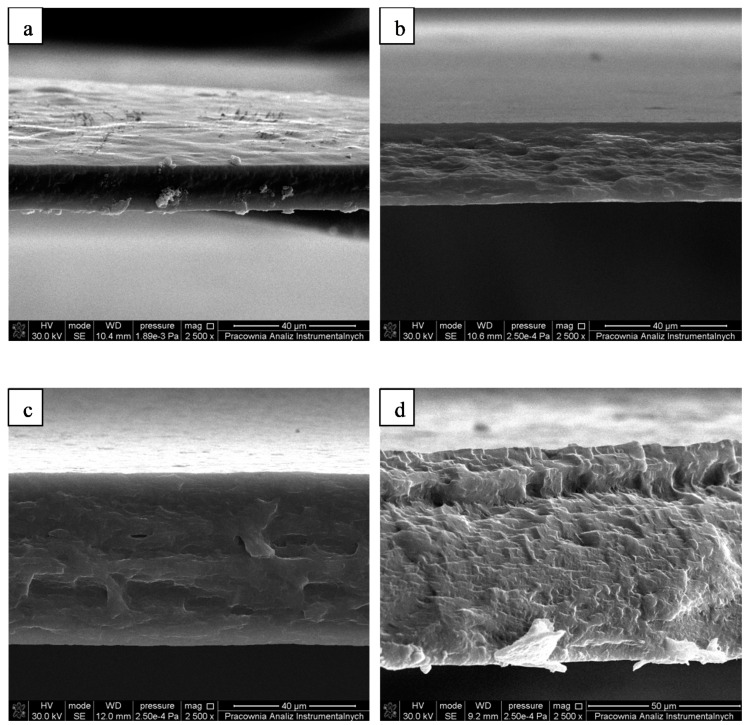
Scanning electron microscopy (SEM) images of materials’ cross-section: (**a**) Coll, (**b**) Coll90/10BG, (**c**) Coll70/30BG, (**d**) Coll50/50BG.

Scanning electron microscopy (SEM) images of a cross-section of collagen and collagen/β-glucan materials are presented in Figure 4. Based on SEM images, dense morphology of collagen/β-glucan materials was observed. The materials were compact and non-porous structure was visible. Moreover, the blends of those two polymers were homogeneous. Layered structure and precipitation were not observed. The cross-section structure was a result of fracturing the materials.

### 3.5. Contact Angle

For polymer materials, the Owens-Wendt method is often used to calculate the surface free energy and its polar and dispersion components. Free surface energy determines the potential application of the material, and its components provide more detailed information about the studied surface [31]. The results of contact angle measurements are shown in Table 3. 

The surface free energy for collagen film was 36.5 mJ/m^2^. Addition of 10% of β-glucan increased the γ value to 41.6 mJ/m^2^. However, a higher amount of β-glucan incorporated into matrices, compared to Coll90/10BG, decreased surface free energy to 38.3 mJ/m^2^. Surface free energy values were the same for 30% and 50% BG addition. The same situation may be observed for the values of the polar component. γ^d^_p_ for collagen was 14.5 mJ/m^2^ and increased for Coll90/10BG to 19.4 mJ/m^2^. However, the value of this parameter decreased to approximately 15 mJ/m^2^ for materials with a higher amount (>10%) of this polysaccharide. The highest polarity among the selected materials was for Coll90/10BG because it has the highest polar component of its surface free energy. The values of dispersive components were similar for all the samples (22–23 mJ/m^2^). Calculation of the γ^d^_p_/γ^d^_s_ ratio makes it easy to tell which material is more hydrophilic. Materials were characterized by the same value of γ^d^_p_/γ^d^_s_ ratio, except for Coll90/10BG, which was the most hydrophilic.

### 3.6. Antioxidant Properties

DPPH assay is an electron transfer-based method which measures the capacity of an antioxidant (hydrogen donator) in the reduction of an oxidant. The DPPH method is frequently used as it allows for quick evaluation of antioxidant properties of samples [32]. The dark purple color of the solution changes to yellow when free radical scavengers exist. Therefore, the antioxidant activity of samples may be evaluated using a spectrophotometer. The ability of materials to scavenge DPPH-free radicals is presented in Table 4. After 1 h of contact, the antioxidant ratio of collagen samples was assessed as 17.7%. For materials with β-glucan, the antioxidant ratio decrease, and values were within the range 12.7–14.5%. 

Phosphorylated (1→3)-β-D-glucan could scavenge DPPH radicals in the dose–effect relationship [32]. Hussain et al. noticed that the dose depended on an increase in DPPH scavenging for gamma-irradiated β-glucan [29]. The antioxidant ratio of yeast β-glucan was 17.3% and after irradiation of β-glucan, the DPPH scavenging increased with the irradiation dose [33].

### 3.7. Swelling Properties 

The swelling ratios of collagen materials modified with β-glucan addition are presented in Table 5. Collagen material without β-glucan addition swelled intensively and the swelling ratio was 425%. The swelling ratio decreases as β-glucan addition increases. The lowest swelling ratio was observed for Coll50/50BG and it reached 232%.

### 3.8. Color Change

The parameter L (lightness) for the control sample was higher than that for the tested film after contact with media (negative values). Prepared materials become darker after contact with liquids. However, the change is not visible with the naked eye. The ISO 11664-4:2008 standard states that ΔE > 5 is noticed as a color change. No change of color was thereby noticed for Coll90/10BG film after NaOH treatment (Table 6). The other materials change color after liquid application. 

### 3.9. In Vitro Blood Biocompatibility

The in vitro blood biocompatibility is commonly used to evaluate the effect of materials on the integrity of the red blood cells (erythrocytes). Damaged erythrocytes release hemoglobin, therefore spectroscopic measurements can be used to detect this [34]. According to the ASTM F756-00 standard, materials with the hemolytic ratio between 0% and 2% are classified as non-hemolytic, while materials with 2–5% are slightly hemolytic and <5% are classified as hemolytic [22]. Based on the above, all the tested materials were classified as non-hemolytic. Collagen showed 0.69% of hemolysis ratio, and with increasing addition of β-glucan into materials, the hemolysis ratio decreased (Table 7). For Coll50/50BG, the recorded absorbance value was lower than for the negative control. Therefore, we believe that Coll50/50BG material may positively affect the stability of blood cells and reduce their disintegration.

### 3.10. Cytocompatibility Studies

**Figure 5:**
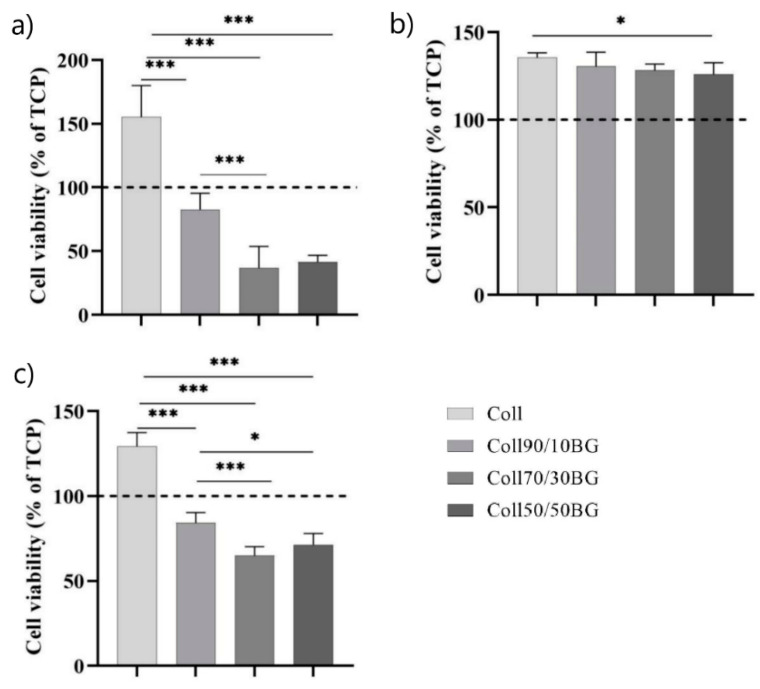
Viability of HaCaT cells (**a**) after 24 h direct culture on the materials, (**b**) after 24 h exposure to the material extracts, and (**c**) after 72 h direct culture on the materials that were “pre-conditioned” for 24 h with culture medium. * *p* ≤ 0.05; *** *p* ≤ 0.001.

The cell viability above 70% is acceptable to regard materials as non-cytotoxic/cytocompatible [35]. Culturing cells for 24 h directly on the fish skin collagen (Coll) increased the viability of HaCaT cells compared to TCP control (Figure 5a). The addition of 10% β-glucan to collagen matrices decreased cell viability vs. pure collagen matrix, but the viability of cells remained close to that on TCP control. In contrast, the addition of higher concentrations of β-glucan (BG) (30% and 50%) to collagen matrices resulted in a marked decrease of cells’ viability (36–41% vs. TCP). Notably, all examined material extracts increased cells’ viability vs. TCP control (Figure 5b), probably due to some collagen-related material degradation products. Cell viability was comparable for all studied extracts except for cells treated with the extracts from Coll50/50BG, suggesting that 50% or higher BG content in the collagen matrix may result in degradation products inhibiting cell growth. Longer 72 h cultures on pre-incubated materials (Figure 5c) showed a similar trend to that obtained after 24 h culture. Direct culture on the BG-enriched materials significantly decreased cell viability vs. pure collagen matrix, but again, viability of cells cultured for 72 h on matrices enriched with 10% BG was close to that on TCP control, whereas higher content of BG in collagen matrix resulted in cell viability below 70% vs. TCP control. Overall, these results suggest that small (i.e., 10%) BG addition to collagen matrix is safe and should allow cells to survive at sufficient number on direct contact with this material. Moreover, the material dissolution products are certainly not toxic and may actually have some beneficial effects on cells.

## 4. Discussion

The mechanical properties of collagen materials improve within the β-glucan concentration. The improvement of mechanical properties after the β-glucan addition was noticed also for other polysaccharides, such as, e.g., starch and arabinoxylans. Starch was mixed with β-glucan in ratios of 75/25, 50/50, and 25/75, and thin films were prepared from the obtained mixture [36]. The increase of maximum tensile force has also been noticed for films with β-glucan addition compared to arabinoxylans-based materials [37]. The increase of mechanical parameters is related to the glucan ability to absorb water, which enhances the material flexibility. It is an important factor which results in the improvement of the properties of the obtained films for their application as wound dressings.

The topography of the prepared materials is homogenous, non-porous, and with the random collagen fiber distribution. The same situation was observed on SEM pictures by Jana et al., where aggregated collagen fibers were observed on cross-linked fish collagen [38]. Also, Kozłowska et al. noticed banded collagen fibrils with 67 nm diameter on the surface of Northern pike collagen film. The topography of their films was much smoother, with Ra = 5.4 nm [39]. Smoother collagen surface was also observed for rat tail tendon collagen films, Ra = 3.64 nm and Rq = 4.59 nm [40]. Tang et al. analyzed the structure of tilapia skin collagen films. They noticed compact, smooth, and homogeneous surface on the upper side of the film, however fibrous structure was observed on the lower surface [41]. Novák et al. prepared one-component β-glucan (isolated from *Sacharomyces cerevisiae*) films [42]. They observed a granular-like structure with a height of the granular particles of about 100 nm. Moreover, as in this work, the material was non-porous. The porous structure is a crucial feature of materials with biomedical applications. However, they noticed that β-glucan allows for water and wound exudates to pass through the material, so porosity, in this case, is not crucial. β-1,3-glucan films were also prepared by Klimek et al. by using the thermal method and by dialysis [43]. The structure of the material prepared by the thermal method was smooth, and on the surface of β-glucan material after the dialysis process, randomly distributed precipitates were observed. Therefore, the process of preparing materials influences the surface structure.

The results obtained for contact angle measurements suggest that there are fewer interactions between the reactive groups of those polymers. Thus, the polar groups of collagen have been hidden below the surface of the film. After the addition of β-glucan into the matrices, the decrease of contact angle for glycerol was observed. Collagen has two types of hydrophilic groups—amine and hydroxyl ones. Also, β-glucan has hydroxyl groups in the structure. The β-glucan addition to collagen results in hydrogen interactions between their functional groups. The addition of a small amount of β-glucan improves the film hydrophilicity as more hydroxyl groups are present compared to pure collagen. The number of interactions increases with an increasing amount of β-glucan. As a result, those groups are blocked and the decrease of polar components, as well as surface free energy, is observed. Lewandowska et al. showed that the addition of chitosan (polysaccharide) into collagen/hyaluronic acid films decreases the contact angle for glycerol [44]. Also, chitosan mixed with collagen increased the contact angle for glycerol compared to collagen alone [40]. Surface free energy calculated by other researchers for collagen films from different sources of collagen were between 31.4 to 38.6 mJ/m^2^. The values of surface free energy were similar to our result (36.5 mJ/m^2^) [31,39,40].

In the case of inflammatory healing, leukocytes in the process of phagocytosis cleanse the wound of dead and damaged cells as well as pathogens. This process leads to the overproduction of free radicals, e.g., peroxide anion, hydrogen peroxides, and hydroxyl anion [45]. The oxygen balance of the cell is maintained by means of enzymes and non-enzymes to maintain the advancement level. However, if the amount of reactive formed is greater than the natural ability to eliminate them, it is said to be an oxidative stress. Excessive levels of free radicals disrupt the control of oxidant/antioxidant cells and cause enzyme inactivation, DNA nuisance, and lipid peroxidation. It leads to the formation of damage to the visible skin wound and prolongs its healing process [46]. The antioxidant properties of carbohydrates are relatively low [47]. Moreover, free-radical scavenging depends on the molecule’s solubility [48]. Sun et al. did not observe the antioxidant properties of oat β-glucan and suggested that poor solubility of β-glucan was the reason for low DPPH scavenging [49]. We think that lower DPPH scavenging after β-glucan addition may be related to the occupation of active hydroxyl groups (hydrogen donors) by hydrogen binding with the -NH_2_ collagen group.

Currently, it is believed that wound dressings should provide a moist wound environment and remove the excess wound exudate [50]. The swelling ratio decreases as β-glucan addition increases. The opposite situation was observed by Sionkowska et al., where after 1 h of incubation in PBS buffer, collagen materials swelled by about 228%, and after 25% polysaccharide addition (chitosan) into materials composition, the swelling ratio increased to 572% [51]. The authors observed the highest swelling ratio after crosslinking collagen/chitosan material with tannic acid (1358%). Collagen scaffold (from fish scale) cross-linked with glutaraldehyde swelled rapidly by about 380% after 30 min of incubation in PBS buffer and continued to swell until 410% was achieved [52]. Khan et al. showed that, depending on the extraction source of β-glucan, swelling properties differed [53]. The β-glucan powder extracted from different species of mushrooms, *Agaricus bisporus*, *Pleurotus ostreatus*, and *Coprinus attrimentarious*, swelled 345%, 374%, and 449%, respectively. However, the methodology used in that article was different than ours [54]. 

The transparent materials allow continuous visualization of the wound bed without removing the dressing [55]. Therefore, the color change after contact with liquids with different pH was performed. Materials change color after HCL and water application, NaOH treatment does not change the color of films. Although an adequate explanation should appear on the packaging, the color change should not hinder the visual inspection of the wound. The significant color change was noticed by Chang after the β-glucan addition to polysaccharide in the form of pullulan [54]. Similar results were obtained by Kurek, where β-glucan was added to the starch [56]. The decrease of the L parameter was noticed as the increase of a and b parameters. It has been assumed that the presence of interactions between polymers and β-glucan plays an important role. We observed that the presence of hydrogen interactions between collagen and β-glucan results in the form of a more yellowish film.

We noticed that blood compatibility of materials with β-glucan was slightly better compared to collagen alone. Tan et al. observed better blood compatibility of collagen after dialdehyde carboxymethyl cellulose addition [57]. They suggest that the lower hemolysis ratio was achieved due to reducing the active group (-NH_2_) by the crosslinking effect. Culturing cells on the *Hypophthalmichthys nobilis*-isolated collagen increased the proliferation of HaCaT cells after 24 h compared to the control (TCP). Zhou et al. showed that the tilapia collagen nanofibers promoted cell adhesion and proliferation, increasing the proliferation rate of HaCat cells to 114% [12]. Hydrogel based on β-glucan allow for drug release and show no cytotoxicity against L929 cells. Furthermore, compared to traditional materials (like gauze, bandages), materials based on β-glucan can provide the moist environment of the wound bed and accelerate the healing process [19]. An in vivo study shows that β-glucan increased the epithelialization and wound contraction in the modified diabetic male mice [58]. In our study, the best biological properties were found for collagen material with cell viability ca. 160% compared to TCP control after 24 h incubation. This is not surprising, as collagen is a natural component of the extracellular matrix of several tissues and enhances cells’ attachment, spreading, and growth. BG addition to such collagen matrix may affect cell growth compared to pure collagen film, but our results show that small BG content is not toxic to the cells on direct contact with such BG-enriched matrices and the degradation products from such BG-enriched materials have similar to pure collagen matrix and beneficial effects on the cells. Thus, Coll90/10BG display optimum parameters to be used for further study as potential drug carriers.

## 5. Conclusions

Collagen from fish can be mixed with β-glucan and prepared in the form of thin films by using the solvent casting method. We have determined that β-glucan and collagen were bound together by a hydrogen bond. It is assumed that the hydrogen bond between the hydroxyl group of β-glucan and amine group of collagen protects materials from showing hemolytic activity. Also, due to this type of bonding, the antioxidant properties of films slightly decrease. β-glucan addition increased roughness of material surfaces and resulted in a more rigid character of the materials. All the materials could swell in vitro, and a small addition of β-glucan provided a more hydrophilic character of the surfaces. Finally, our cytotoxicity studies indicated that BG can be safely added at small (10% or less) quantities to collagen matrix, they sufficiently support cell growth, and the dissolution products of such matrices may actually provide some beneficial effects to the surrounding cells/tissues. 

## Figures and Tables

**Table 1:** Assignments of the major frequencies of collagen, β-glucan, and collagen/β-glucan material.

Specimen	Assignments	Wavenumber (cm^−1^)
Coll	Amide A	3315
	Amide B	3049
	Amide I	1618
	Amide II	1527
	Amide III	1225
BG	C–C, C=O	928–1200
	C–O–C	1014
	β glycosidic bonds	896
Coll90/10BG	Amide A	3292
	Amide B	2928
	Amide I	1634
	Amide II	1531
	Amide III	1222
	C-O-C	1007
	β glycosidic bonds	898

**Table 2:** Surface roughness parameters for different kinds of films.

Specimen	Rq (nm)	Ra (nm)
Coll	33.4	26.2
Coll90/10BG	36.5	29.2
Coll70/30BG	59.0	47.8
Coll50/50BG	125	100

**Table 3:** Surface free energy (γ) and its polar (γ^d^_p_) and dispersive (γ^d^_s_) components for collagen materials modified with β-glucan.

Specimen	θ_G_ (°)	Θ_D_ (°)	γ (mJ/m^2^)	γ^d^_p_ (mJ/m^2^)	γ^d^_s_ (mJ/m^2^)	γ^d^_p_/γ^d^_s_
Coll	61.90 ± 1.15	57.43 ± 0.98	36.51 ± 0.37	14.46 ± 0.17	22.05 ± 0.20	0.65
Coll90/10BG	53.33 ± 0.90	54.43 ± 0.59	41.61 ± 0.26	19.41 ± 0.14	22.20 ± 0.12	0.87
Coll70/30BG	59.58 ± 0.70	54.70 ± 0.18	38.33 ± 0.13	15.11 ± 0.08	23.22 ± 0.04	0.65
Coll50/50BG	59.60 ± 0.10	54.54 ± 0.28	38.36 ± 0.09	15.03 ± 0.03	23.33 ± 0.06	0.64

**Table 4:** Antioxidant ratio of collagen material modified with β-glucan.

Specimen	Antioxidant Ratio (%)
Coll	17.7 ± 1.5
Coll90/10BG	14.5 ± 1.3
Coll70/30BG	13.8 ± 0.7
Coll50/50BG	12.7 ± 0.4

**Table 5:** Swelling ratio of collagen materials with β-glucan addition.

Specimen	Swelling Ratio (%)
Coll	425.3 ± 41.1
Coll90/10BG	315.2 ± 33.4
Coll70/30BG	237.8 ± 33.8
Coll50/50BG	232.8 ± 5.8

**Table 6:** Color parameters (L—lightness, a—red (+) to green (−), b—yellow (+) to blue (−b), and ΔE—total color difference) of the materials.

Specimen	Color Parameter	Solutions		
		HCl	NaOH	Water
Coll	L	−10.48 ± 2.11	−7.91 ± 1.78	−9.09 ± 0.92
	A	0.41 ± 0.09	0.70 ± 0.14	1.52 ± 0.19
	B	2.97 ± 0.73	1.84 ± 0.25	2.97 ± 0.47
	ΔE	10.90 ± 1.03	8.15 ± 0.92	9.68 ± 0.37
Coll90/10BG	L	−7.82 ± 0.32	−3.36 ± 0.19	−12.18 ± 1.23
	A	0.73 ± 0.11	0.37 ± 0.07	1.41 ± 0.09
	B	1.53 ± 0.28	2.72 ± 0.16	3.49 ± 0.38
	ΔE	7.99 ± 0.32	4.33 ± 0.06	12.75 ± 0.59
Coll70/30BG	L	−12.07 ± 1.07	−10.06 ± 0.71	−12.76 ± 1.41
	A	1.14 ± 0.09	1.25 ± 0.04	0.51 ± 0.08
	B	2.10 ± 0.17	4.74 ± 0.62	2.29 ± 0.71
	ΔE	12.30 ± 0.54	11.18 ± 0.36	12.97 ± 0.66
Coll50/50BG	L	−13.00 ± 1.26	−12.00 ± 0.92	−10.24 ± 1.02
	A	1.08 ± 0.12	−0.40 ± 0.02	1.26 ± 0.03
	B	3.26 ± 0.22	4.40 ± 0.77	2.41 ± 0.71
	ΔE	13.44 ± 0.63	12.79 ± 0.48	10.59 ± 0.51

**Table 7:** Hemolysis ratio calculated for collagen and collagen/β-glucan films.

Specimen	Hemolysis Ratio (%)
Coll	0.69 ± 0.03
Coll90/10BG	0.20 ± 0.09
Coll70/30BG	0.19 ± 0.04
Coll50/50BG	0 *

* measured values of absorbance for the material were lower than for the negative control.

## Data Availability

The data presented in this study are available on request from the corresponding author.
